# Predicting colorectal cancer risk from adenoma detection via a two-type branching process model

**DOI:** 10.1371/journal.pcbi.1007552

**Published:** 2020-02-05

**Authors:** Brian M. Lang, Jack Kuipers, Benjamin Misselwitz, Niko Beerenwinkel

**Affiliations:** 1 Department of Biosystems Science and Engineering, ETH Zurich, Basel, Switzerland; 2 SIB Swiss Institute of Bioinformatics, Basel, Switzerland; 3 Department of Visceral Surgery and Medicine, Inselspital Bern and Bern University, Bern, Switzerland; 4 Department of Gastroenterology and Hepatology, University Hospital Zurich and Zurich University, Zurich, Switzerland; University of California Irvine, UNITED STATES

## Abstract

Despite advances in the modeling and understanding of colorectal cancer development, the dynamics of the progression from benign adenomatous polyp to colorectal carcinoma are still not fully resolved. To take advantage of adenoma size and prevalence data in the National Endoscopic Database of the Clinical Outcomes Research Initiative (CORI) as well as colorectal cancer incidence and size data from the Surveillance Epidemiology and End Results (SEER) database, we construct a two-type branching process model with compartments representing adenoma and carcinoma cells. To perform parameter inference we present a new large-size approximation to the size distribution of the cancer compartment and validate our approach on simulated data. By fitting the model to the CORI and SEER data, we learn biologically relevant parameters, including the transition rate from adenoma to cancer. The inferred parameters allow us to predict the individualized risk of the presence of cancer cells for each screened patient. We provide a web application which allows the user to calculate these individual probabilities at https://ccrc-eth.shinyapps.io/CCRC/. For example, we find a 1 in 100 chance of cancer given the presence of an adenoma between 10 and 20mm size in an average risk patient at age 50. We show that our two-type branching process model recapitulates the early growth dynamics of colon adenomas and cancers and can recover epidemiological trends such as adenoma prevalence and cancer incidence while remaining mathematically and computationally tractable.

## Author summary

Colorectal cancer is a major public health burden. The development of colorectal cancer starts with the mutational initiation of non-cancerous growths in the form of benign adenomatous polyps. These adenomas grow over time with the potential to develop into carcinomas. Many mathematical and simulation-based models have been used to gain insight into this process. We aimed to understand rates of adenoma growth and transition into carcinomas, to enable better planning of colorectal cancer screening strategies. To this end, we expand the two-type branching process model, and fit it on data describing the frequency of sizes of adenomas and carcinomas. The results provide new, data-based, estimates of the rates of development for colorectal cancer.

## Introduction

Within the intestinal epithelium, the crypts of the colon house stem cells populate and maintain one of the most dynamic cell populations in humans. It is within this high-turnover environment that spontaneous colorectal cancer (CRC) may gain its start. CRC develops via precancerous adenomatous polyps that reside in the colon for several years. Transition from stem cell to adenoma is accompanied by several somatic mutations, typically involving complete mutational inactivation of the Adenomatous Polyposis Coli (*APC*) tumor suppressor gene [1] or mutations disrupting β-catenin function [2]. Transition from an adenoma to a cancerous phenotype can often be attributed to acquisition of chromosomal instability and mutational events in tumor suppressor genes such as *KRAS*, *TP53*, or the *SMAD2* and *SMAD4* genes in the transforming growth factor (TGF-β) pathway [1].

While most adenomas will not progress to carcinoma, colorectal carcinoma (CRC) is still the 2^nd^ leading cause of cancer-related mortality in the United States with 50,260 deaths in 2017 [3]. Because some adenomas may eventually develop into malignant tumors, screening strategies seek to discover and remove these lesions prior to cancer transition. Several screening approaches using endoscopy or biomarkers for detection and removal of adenomas and/or early detection of CRC were demonstrated to reduce CRC-related mortality [4–7]. However, colonoscopy, which visualizes the whole colon and remains the diagnostic gold standard for adenoma and carcinoma detection, has not been tested in randomized controlled trials [8, 9]. Most industrialized countries have already implemented recommendations for screening based on clinical observations and computational models that utilize real world observational data and data from randomized controlled trials [10–13]. However, the design of optimal population-level screening strategies is an unmet need in clinical gastroenterology.

Current recommendations are based on large simulation-based computational models of populations (microsimulations). However, since crucial information, i.e., the distribution of growth and transition rates between adenomatous polyps and cancer is lacking, these models rely heavily on parameter assumptions [14–16]. For instance, the average time an adenoma will reside in the colon and can be removed (CRC screening window) is unknown. These parameters cannot be determined experimentally, due to the risks of leaving adenomas in situ and potential side effects associated with colonoscopy [12].

As a complementary approach to microsimulation, mathematical models with simplifying assumptions have been used to test hypotheses about the dynamics that generate colorectal carcinomas. One such collection of models, the multistage framework established by Armitage and Doll, suggests that cancer is not generated by a single spontaneous event, but rather the product of a sequence of rate-limiting events [17]. The number of these rate-limiting steps were estimated through the examination of the incidence of various cancers at each age [17, 18]. The two-stage model of carcinogensis moved to a slightly more complex formulation, allowing for certain events in the development of cancer to affect the net growth rate of transformed cells [19–21]. This two stage model has been generalized to *k*-stages, allowing for a collection of rate-limiting steps prior to an eventual clonal expansion in the (*k* − 1)^th^ stage [22–25]. While previous research stayed firmly in the realm of incidence of cancer, subsequent work on the multistage model of carcinogensis has produced expressions for the number and size distributions of growths in the clonally expanding cell type [26].

Parting from the multistage clonal expansion model (MSCE) of carcinogenesis as described by Moolgavkar, Luebeck and others [20–24, 26] there are similarly named multitype branching process models which can be applied to cancer development [27]. These models describe the frequency distributions of cell types over time and are useful to investigate a broad range of hypotheses for CRC development within the framework of multistage carcinogenesis. The use of multitype branching processes involves the definition of a finite number of cell types (e.g. stem cells, adenoma cells, cancer cells) and the stochastic transition probabilities between cell types determining growth dynamics. In comparison to the MSCE described previously, these models often allow birth and death events at each stage. Classically, branching process models have been used to examine rates of appearance and extinction for each cell type [28, 29] but are now used regularly to examine many biological processes, such as the development of ovarian cancer [30], drug resistance in pancreatic, colorectal, and melanoma cancers [31], lung cancer screening timelines [32–34], genetic heterogeneity in cancers [35], as well as the general demonstration that branching processes can recapture population dynamics of cancer development, intratumor heterogeneity and generation of metastasis [27].

Despite significant progress in mathematical modeling of CRC, important open questions remain. While there are numerous models of CRC growth [36], it is rare for the models to be mathematically solved to the point of exact calculations of probability distributions for the number of cells of each cell type. Furthermore, in cases where probability distributions were calculated, they are typically reliant on strong parameter assumptions that limit the opportunity to truly estimate parameters and their uncertainty (i.e. parameter inference). Therefore it remains difficult to assess the transition rates between cell types that underlie cancer progression. In CRC, parameter inference would provide strong evidence for the rates determining average-risk CRC development and enable more accurate simulation-based predictions of optimal screening timelines.

**Fig 1:**
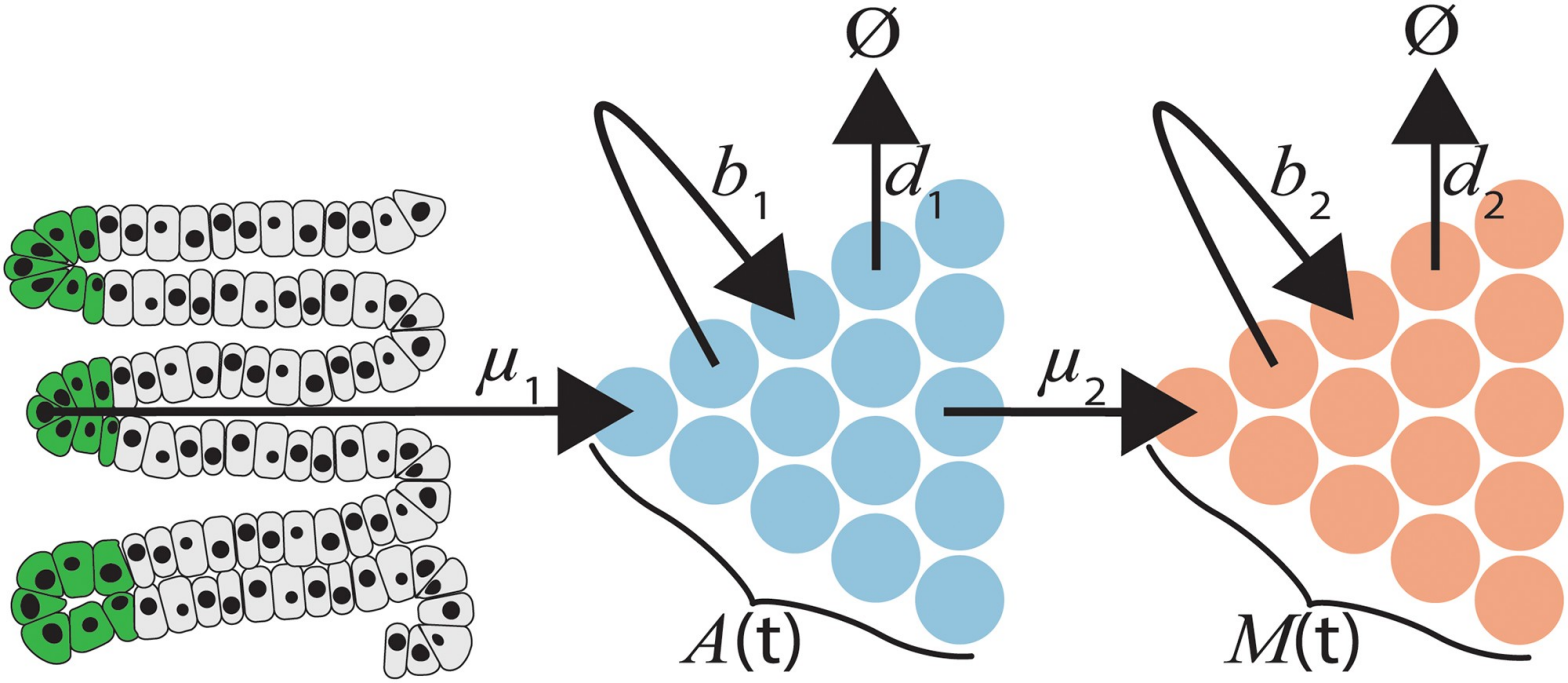
Two-type branching process model of colorectal cancer progression. Cells immigrate from a static population of colonic crypt stem cells (green cells) into the adenoma compartment *A* with rate *μ*_1_. Compartment *A* grows with rate *b*_1_ and decreases with rate *d*_1_. With rate *μ*_2_ adenoma cells generate malignant cells, *M*. Cancer compartment *M* grows with rate *b*_2_ and decreases with rate *d*_2_. The total number of cells in compartments *A* and *M* and time *t* are denoted *A*(*t*) and *M*(*t*), respectively.

Here we build upon the two-type branching process model described by Antal and Krapivsky [37]. We consider the initiation, birth, and death processes that generate the observable quantities of CRC natural history, namely adenoma prevalence and cancer incidence, in the context of this two-type branching process model (Fig 1). The initiation, birth, and death of cells in the adenoma compartment (*A*) represent and encompass all processes that affect adenoma development in average risk patients, while the transition of cells from compartment *A* into the carcinoma compartment *M* represents and encompasses all processes that could lead to the malignant transformation of adenomatous polyp cells. We derive a new approximation which enables computation of the age-specific size distributions of colorectal cancers as well as allowing for model identifiability and parameter inference. Through the fitting of our model to epidemiological data from the Clinical Outcomes and Research Initiative (CORI) endoscopic procedure database, as well as the Surveillance Epidemiology and End Results (SEER) cancer registry, we provide estimates of colorectal cancer growth rates and provide model-based evidence for the natural history of colorectal cancer development.

## Materials and methods

### CORI adenoma prevalence data

The Clinical Outcomes Research Initiative (CORI) National Endoscopic Database (NED) V3 and V4 are clinical databases of endoscopic procedures completed in the US from 1995 to 2015 [38]. Each observation comprises a single endoscopic procedure as well as demographic data about the individual on which the procedure was carried out. CORI procedure data includes colonoscopy findings such as endoscopist-reported longest adenoma dimension millimeter. We group adenoma size into mm-size bins and convert these sizes to cell-numbers assuming 1 cm^3^ corresponds to approximately 10^8^ cells [39] and a half-ellipsoid shape as described in the S1 Appendix Eq. 54. For this work, we select all colonoscopies undertaken on average risk patients with no prior colonoscopy history. For our final model fitting, we include 8,124 procedures from CORI V3 with adenoma detection as well as normal colonoscopy findings in the age group 40 to 49 years. Our model assumes exponential growth of adenomas, and for this reason we limit ourselves to patients younger than 50 years of age, where we still observe an age-size relationship (Figure A in S1 Appendix).

### SEER cancer incidence data

The Surveillance Epidemiology and End Results (SEER) research database comprises cancer incidence and at-risk population data in the US from 1973 to 2014 [40]. Each observation is composed of a single tumor observation with patient demographic information (sex, race, age, and calendar year) as well as endoscopist-reported largest carcinoma dimension in mm. Similar to our procedure for adenomas (see above) we group carcinoma size into mm-size bins (.5 mm on either side of reported size) and convert to cell number assuming 1 cm^3^ corresponds to approximately 10^8^ cells [39] and a half-ellipsoid shape as described in S1 Appendix Eq. 54. For fitting compartment *M* to SEER data, we include 54,835 tumor size observations indicated as ICD-O-3 codes 18. (0-9) for ages 40 through 60 and incidence data from ages 40 through 60 (114,595 observations across 7,399 year, sex, registry, and age groupings).

Two forms of censoring in the SEER data will affect observed carcinoma size: Firstly, individuals with very small cancers (<0.5 mm in size) will either be asymptomatic or the carcinoma will be missed due to small size. Secondly, since symptoms are strongly associated with carcinoma size, individuals with large tumors will preferentially undergo diagnostic evaluations resulting in censoring of large and very large CRC. The SEER database provides information regarding incident cancer cases as well as number of at-risk individuals at each age.

From the SEER database we know I(*t*), the number of incident cancer cases at age *t*, and R(*t*), the number of at-risk individuals at age *t*, but we do not know P(*t*), the prevalent cancer cases at age *t* who were previously incident cancer cases, since these are censored in the data. We therefore want to estimate the number of prevalent cases, P(*t*). If we assume that these are the three possible conditions for an individual we define the non-normalized total population size as

$$\begin{array}{c} T(t)=R(t)+I(t)+P(t). \end{array}$$

To be able to estimate prevalent cases with varying population sizes, we standardise our population by dividing by the total population size. We therefore define a normalized population denoted with carets:

$$\begin{array}{c} 1=\hat{R}\left(t\right)+\hat{I}\left(t\right)+\hat{P}\left(t\right), \end{array}$$

where $\hat{R}\left(t\right)$ is the proportion of at-risk individuals at age *t*, $\hat{I}\left(t\right)$ is the proportion of newly incident cancers at age *t* and $\hat{P}\left(t\right)$ is the proportion of individuals with previously diagnosed cancers at age *t*. The standard calculation of age-specific incidence rate is the ratio between incident cancers at a given age and the total at-risk population size for that age, $\frac{I(t)}{R(t)+I(t)}$. At each time step, this fraction of the previous at-risk group moves to the incident group, and we can recursively calculate $\hat{R}\left(t\right)$ with the iterative formula

$$\begin{array}{c} \hat{R}\left(t\right)=\hat{R}\left(t-1\right)-\left(\hat{R}\left(t-1\right)\frac{I(t)}{R(t)+I(t)}\right) \end{array}$$ (1)

with $\hat{R}\left(t=0\right)=1$, and where we use the fact that $\frac{I(t)}{R(t)+I(t)}=\frac{\hat{I}\left(t\right)}{\hat{R}\left(t\right)+\hat{I}\left(t\right)}$ does not depend on the total population size. The normalized proportion of incident cancers is simply

$$\begin{array}{c} \hat{I}\left(t\right)=\hat{R}\left(t-1\right)-\hat{R}\left(t\right) \end{array}$$ (2)

while the normalized proportion of prevalent cases is

$$\begin{array}{c} \hat{P}\left(t\right)=1-\hat{R}\left(t-1\right). \end{array}$$ (3)

Then, to estimate P(*t*), we rely upon the correspondence between the normalized and non-normalized populations $P\left(t\right)=\hat{P}\left(t\right)T\left(t\right)$, $I\left(t\right)=\hat{I}\left(t\right)T\left(t\right)$. By rearranging the latter, we estimate the unknown total population size $T\left(t\right)=I\left(t\right)/\hat{I}\left(t\right)$ from the known real-life values from the SEER data I(*t*), and by substituting into the former obtain

$$\begin{array}{c} P\left(t\right)=I\left(t\right)\frac{\hat{P}\left(t\right)}{\hat{I}\left(t\right)} \end{array}$$ (4)

For example, from the SEER data we have I(*t* = 50) = 3,218 newly incident cancer cases at age 50, and can compute the number of censored, prevalent cancers, $P\left(50\right)=I\left(50\right)\frac{\hat{P}\left(50\right)}{\hat{I}\left(50\right)}$. The normalised fraction of prevalent cases was estimated from the SEER data to be $\hat{P}(50)=0.00284$. The normalized value of $\hat{I}\left(50\right)=0.000491$. leads to a prevalent-to-incident ratio of $\hat{P}\left(50\right)/\hat{I}\left(50\right)=5.795$, so that the 3,218 incident cases mean that we impute P(50) = 18,651 prevalent cases for age 50. These are placed in a bin corresponding to growths larger than 40 mm, the median growth size reported across our data.

### Two-type branching process model

A two-type branching process model with immigration is used to model the stochastic dynamics of colorectal cancer development in an average-risk US population. In this model we have two cell types, *A* and *M* which relate to adenomatous and malignant cells, respectively (Fig 1). The dynamics are as follows:

$$\begin{array}{ccc} \emptyset & \overset{\mu_{1}}{\to } & A\\ A & \overset{b_{1}}{\to } & A+A\\ A & \overset{d_{1}}{\to } & \emptyset \\ A & \overset{\mu_{2}}{\to } & M\\ M & \overset{b_{2}}{\to } & M+M\\ M & \overset{d_{2}}{\to } & \emptyset \end{array}$$

#### Compartment *A*

Compartment *A* can be solved (S1 Appendix Eq. 15) for the probability *P*_*t*_(*A*(*t*) = *k*) of having *k* type-*A* cells at age *t* by setting *μ*_2_ = 0:

Pt(A(t)=k)=(1-p)r(r+k-1k)pk (5)

which is a negative binomial distribution describing the probability *p* of real-valued *r* failures given *k* successes with parameters $r=\frac{\mu_{1}}{b_{1}}$, $p\left(t\right)=\frac{b_{1}\left(e^{\gamma_{1}t}-1\right)}{\left(b_{1}e^{\gamma_{1}t}-d_{1}\right)}$, and *γ*_1_ = *b*_1_ − *d*_1_. For adenoma observation *t* at a given age with a binned size, we define $O_{i}^{A}=\left(L_{i}^{A},\;U_{i}^{A},\;t_{i}^{A}\right)$, where we have a Compartment- *A* observation with lower size bound $L_{i}^{A}$ and upper size bound $U_{i}^{A}$, found at age $t_{i}^{A}$. The likelihood of the parameters Θ^*A*^ = (*μ*_1_, *b*_1_, *d*_1_), given an individual observation $O_{i}^{A}$ is

$$\begin{array}{c} L\left(\Theta^{A}|O_{i}^{A}\right)=I_{p}\left(L_{i}^{A}+1,r\right)-I_{p}\left(U_{i}^{A}+1,r\right) \end{array}$$ (6)

where I_*p*_(*k*, *r*) is the regularized incomplete beta function defined as the ratio of the incomplete beta function B(*p*, *k*+ 1, *r*) over the complete beta function B(*k* + 1, *r*). The latter is the cumulative distribution function (CDF) of the negative binomial distribution.

#### Compartment *M*

For the full model, we modify the previous result from Antal and Krapivsky [37] for the probability generating function to include steady influx into Compartment *A*. From the final generating function *G*(*s*, *t*) (S1 Appendix Eq. 40) we can extract the cumulative probability of having up to *N* cells in Compartment *M* with the residue

$$\begin{array}{c} P\left(M\left(t\right)\leq N|\Theta ,t\right)=\frac{1}{2\pi i}\oint \frac{1}{s^{N+1}}\frac{G(s,t)}{\left(1-s\right)}ds \end{array}$$ (7)

with model parameters Θ = (*μ*_1_, *b*_1_, *d*_1_, *μ*_2_, *b*_2_, *d*_1_). To evaluate the contour integral we develop a large *N* approximation as in [41]. First we rewrite the integral as

$$\begin{array}{c} P\left(M\left(t\right)\leq N|\Theta ,t\right)=\frac{1}{2\pi i}\oint e^{V(s)}ds \end{array}$$ (8)

with:

$$\begin{array}{c} V(s)=-\log(1-s)+\log(G(s,t))-(N+1)\log(s) \end{array}$$ (9)

and evaluate the integral at its saddle point using the stationary phase approximation. This involves solving *V*′(*s*) = 0 and substituting the solution s*,

$$\begin{array}{c} P\left(M\left(t\right)\leq N|\Theta ,t\right)\approx \frac{e^{V(s^{*})}}{2\pi }{\left(\frac{2\pi }{V^{\prime \prime }\left(s^{*}\right)}\right)}^{\frac{1}{2}} \end{array}$$ (10)

Similar to the compartment- *A* case, for a carcinoma finding at a given age with a binned size, we define $O_{i}^{M}=\left(L_{i}^{M},U_{i}^{M},t_{i}^{M}\right)$. Furthermore, the likelihood of the parameters Θ = (*μ*_1_, *b*_1_, *d*_1_, *μ*_2_, *b*_2_, *d*_2_), given an individual observation $O_{i}^{M}$ is

$$\begin{array}{c} L\left(\Theta |O_{i}^{M}\right)\approx P\left(M\left(t\right)\leq U_{i}^{M}|\Theta \right)-P\left(m\left(t\right)\leq L_{i}^{M}|\Theta \right) \end{array}$$ (11)

#### Compartment- *M* extinction given compartment *A* size

We derive the conditional probability of having *k* cells in compartment *A* at time *t* given compartment *M* is empty, *P*(*A*(*t*) = *k*|*M*(*t*) = 0) and use Bayes’ theorem to compute the probability of 0 cells in Compartment *M* given Compartment *A* is of a certain number of cells (S1 Appendix Eq. 53).

#### Modification for λ

When we allow for a resistant sub-population of proportion λ we then have a mixture model which leads to a modification which applies to the likelihoods seen in Eqs 6 and 11:

$$\begin{array}{cc} L(\Theta ,\lambda |O_{i})= & \left\{ \begin{array}{cc} \lambda +\left(1-\lambda \right)L\left(\Theta |O_{i}\right)\; & \text{if}\;0\in (L_{i},U_{i}),\\ \left(1-\lambda \right)L\left(\Theta |O_{i}\right)\; & \text{otherwise} \end{array}\right. \end{array}$$ (12)

#### Complete model

To combine the likelihoods of compartment *A* and *M*, we define a composite likelihood of the model parameters given size data pertaining to both adenomas and malignant cancers. Computed in log space we have:

$$\begin{array}{c} \ell \left(\Theta |O^{A},O^{M}\right)\approx \sum_{i=1}^{K^{A}}\ell \left(\Theta^{A}|O_{i}^{A}\right)+\sum_{j=1}^{K^{M}}\ell \left(\Theta |O_{j}^{M}\right) \end{array}$$ (13)

where *K*^*A*^ and *K*^*M*^ are the number of adenoma and malignant cancer observations respectively.

### Simulations

For validation, we used the Gillespie algorithm as implemented in the R package SSAR [42] to generated stochastic simulations of the two-type branching process model with a set of biologically and computationally feasible parameters. Chosen to reflect cellular dynamics of colorectal cancer development, each parameter defines the per-year rate at which an event can occur within a cell. We chose the following biologically reasonable parameters: *μ*_1_ = 3.1, *b*_1_ = 9, *d*_1_ = 8.8, *μ*_2_ = 10^−5^, *b*_2_ = 9.2, and *d*_2_ = 8.8. The value of *μ*_1_ is chosen with the following simplifying assumptions: an average of 10^7^ colonic crypts with six stem cells per crypt are replaced at an average rate of .2 per day [43], and the average somatic mutation rate per base pair per division is taken to be 2.8 × 10^−9^ [44]. We further assume a 250bp genomic region could trigger an adenoma transition (for example the region of the *APC* gene which is typically mutated in CRC is around this size [45]). Taken together, this leads to a mutation rate of 3,100 per year per individual. Recognizing that inactivation of the tumor suppressor gene APC involves two hits, we multiply this mutation rate by 1/1000 to roughly mimic the second hit. We take rates *b*_1_ and *d*_1_ from Herrero-Jimenez et al. [46] and others who have estimated a birth rate, *b*_1_ of 9 per year and a net-growth rate, *γ*_1_ = *b*_1_ − *d*_1_, of around .18 per year [22, 46]. Compartment- *M* parameters *b*_2_ and *d*_2_ are chosen assuming a doubling of net growth rate for the cancer compartment, while *μ*_2_ is chosen largely for computational convenience, small but large enough to generate growths in our desired age ranges. We simulated this process for 100,000 individuals and uniformly assessed the runs at times up to 80 years. To cope with the real-life phenomena of adenoma-free individuals, we introduce a parameter λ which represents the proportion of individuals in our population who do not develop adenoma at all. For purposes of parameter-recapture in the simulated data, we set λ equal to 45% and attempt to recover this parameter as well.

### Model fitting

We fit the model parameters on the simulated and real data by maximizing likelihoods described in the methods (Eqs 6, 11 and 13) via Nelder-Mead optimization, a numerical optimization algorithm for nonlinear functions, and assess the agreement between stochastic simulation and our model for each compartment by comparing the model-predicted CDF function with the empirical CDF of simulated sizes [47]. Subsequently, we assess parameter uncertainty for our parameter estimates via adaptive Markov chain Monte Carlo (MCMC) for 10,000 steps with a target acceptance rate of 30% [48]. To better illustrate the compartment- *M* likelihood landscape, we perform a grid search. For model fitting we use the parameters *μ*_1_, $\frac{\mu_{1}}{b_{1}}$, *γ*_1_ = *b*_1_ − *d*_1_, *μ*_2_, and *γ*_2_ = *b*_2_ − *d*_2_ to more efficiently search the space. For the simulated data, we perform parameter inference three times: once on compartment *A* only, once on compartment *M* only, and a third time on both compartments simultaneously and considering λ. For the real data, we perform parameter on both compartments simultaneously (Eq 13). We allow for a adenoma-resistant population λ of 55%, chosen after examining the CORI data and determining the maximal adenoma prevalence across all ages (45%) and restrict *d*_1_ = *d*_2_ as a simplifying assumption.

#### Prior on *μ*_1_

We add a prior on the coefficient *μ*_1_ in order to encourage the fit to biologically feasible levels of adenoma initiation. The prior distribution of *μ*_1_ is taken to be lognormal with a mean of 3,100 and a standard deviation of 1/25. This value corresponds to the expected number of mutational events during a year at a given base pair occurring during mitosis in a stem cell of the colonic crypt (see Simulations).

#### Prior on *b*_1_

We utilize a prior for growth coefficient *b*_1_ in order to encourage the fit to biologically feasible levels of adenoma growth. The prior distribution of *b*_1_ is taken to be lognormal with a mean of 9 and a standard deviation of 1/3 [46].

### Ethics statement

The Ethics Commission of the Executive Board of ETH approved this research (EK 2017-N-47).

## Results

### Overview

We developed an extension to the two-type branching process model with adenoma initiation (immigration into compartment *A*) and applied it to the question of colorectal cancer growth dynamics (Fig 1). Previous work established an exact solution to this overall process, but an analytical solution to the size distribution of carcinoma cells at time *t*, *M*(*t*), was not provided [37]. We derive a large-size approximation to the size distribution of compartment *M* (carcinoma cells) and demonstrate its fit on simulated as well as real data from several sources.

In the two-type branching process model, initiation of *A* cells occurs at rate *μ*_1_. These *A* cells then proliferate with rate *b*_1_, die with rate *d*_1_, and transition into *M* cells with rate *μ*_2_. Subsequently, these *M* cells proliferate with rate *b*_2_ and die with rate *d*_2_. Taking into account the occurrence of patients who will not develop adenomas, we allow for a resistant population of λ%. For the purposes of fitting this model on real data, we take the *A* cells to be adenoma cells and the *M* cells to be cells of malignant tumors.

### Validation of the mathematical approximation of our model by simulation

**Fig 2:**
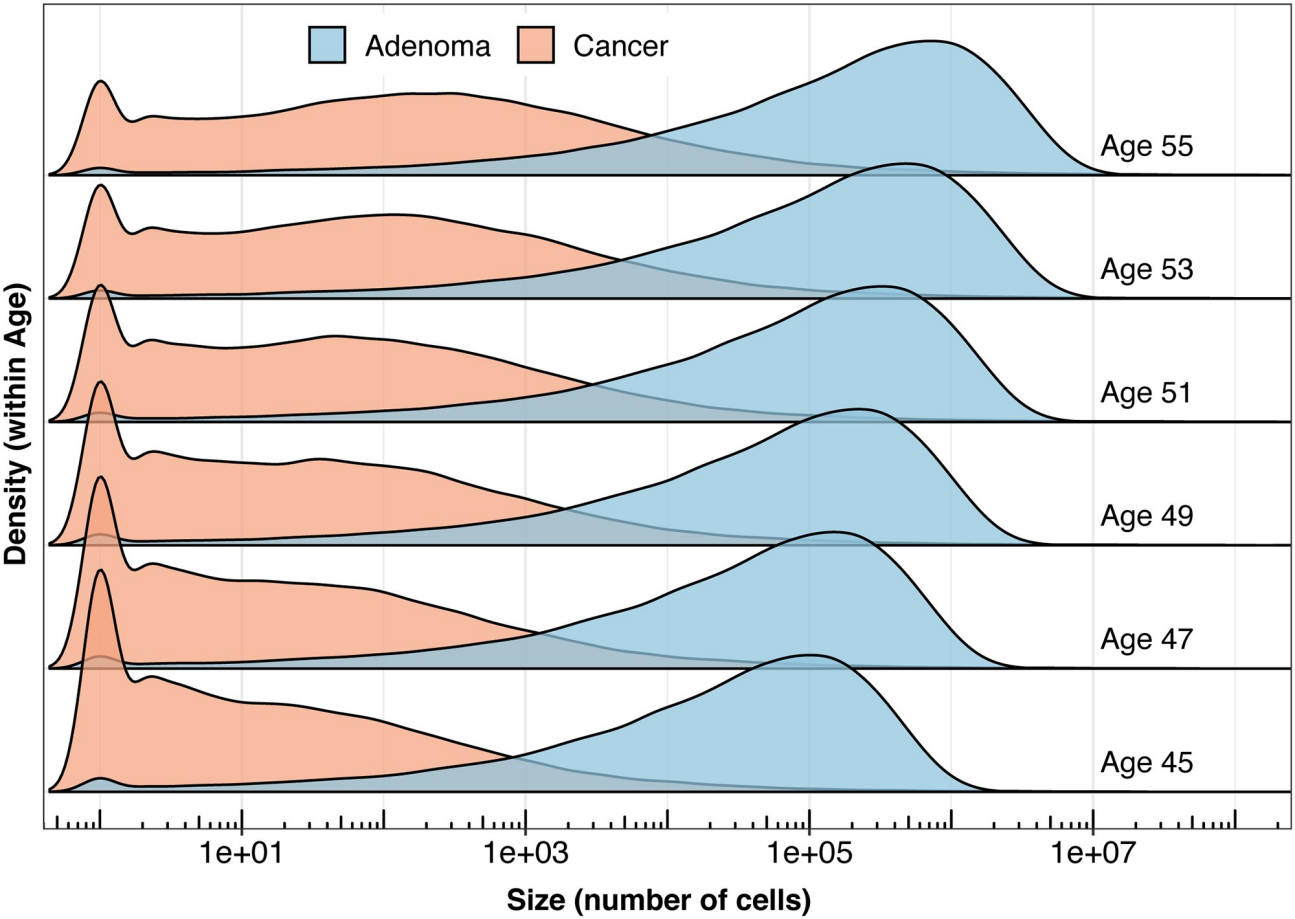
Illustration of simulated data for size of compartments *A* and *M*. We performed 100,000 simulations of the two stage branching process with biologically motivated parameters (*μ*_1_ = 3.1, *b*_1_ = 9, *d*_1_ = 8.8, *μ*_2_ = 10^−5^, *b*_2_ = 9.2, and *d*_2_ = 8.8). Presented are the empirical densities of compartments *A* and *M*, given non-extinction. Heights indicate the density of the size distribution at each given age.

To validate our mathematical approximation, we simulate adenoma and carcinoma growths via stochastic simulation of the two-type branching process (Fig 2) and fit the model to the simulated data. We fit the model by computing the maximum likelihood parameters with our likelihood functions (Eqs 6, 11 and 13). For the simulated data, we present two separate fitting strategies. In the first we demonstrate recovery of biologically-motivated simulated parameters (*μ*_1_ = 3.1, *b*_1_ = 9, *d*_1_ = 8.8, *μ*_2_ = 10^−5^, *b*_2_ = 9.2, *d*_2_ = 8.8) in each compartment separately (Eqs 6 and 11). In the second, we illustrate that we can recapture the simulated parameters by combining the likelihoods (Eq 13), and performing adaptive MCMC.

**Fig 3:**
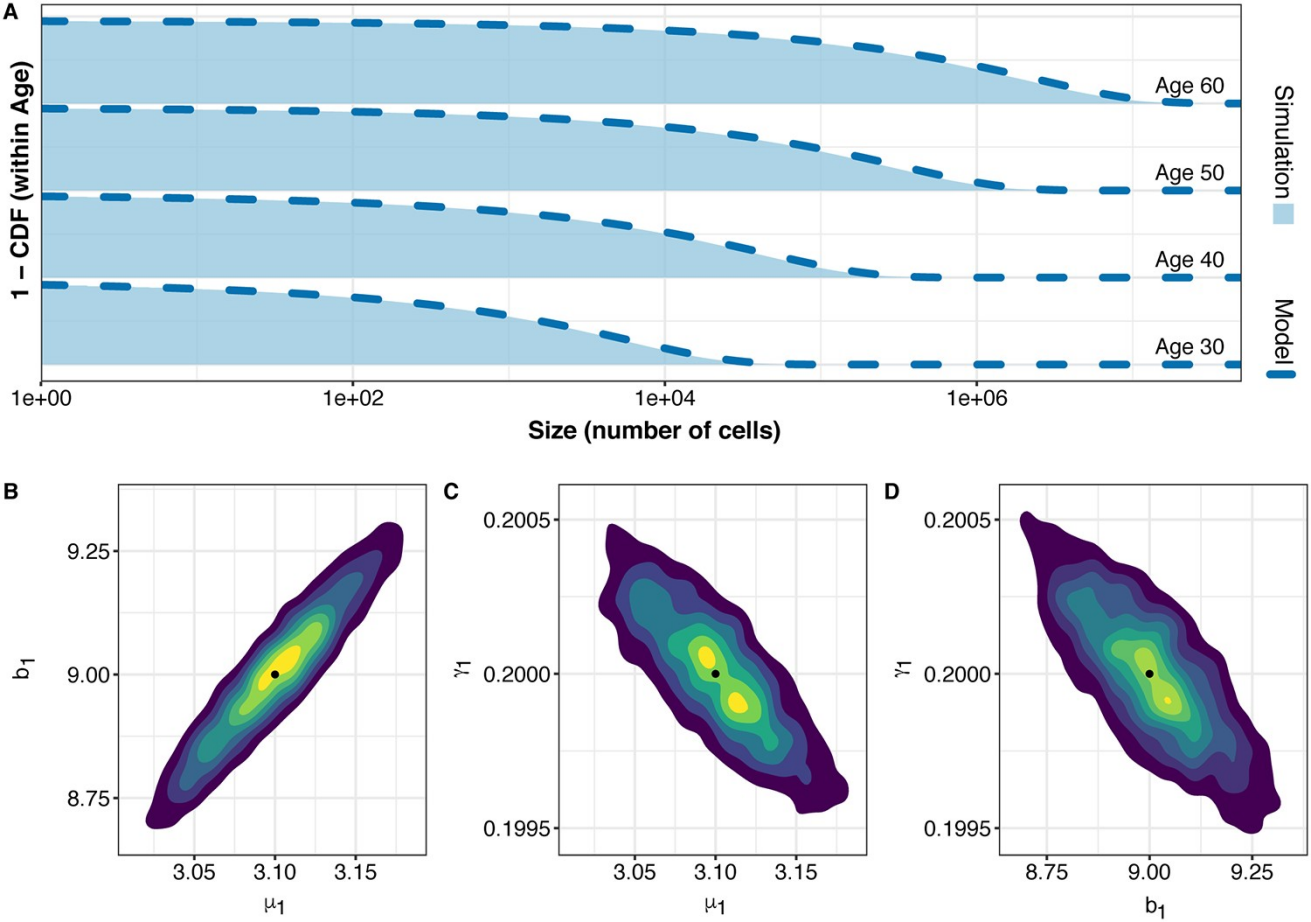
Agreement of simulation and mathematical model for compartment *A*. (A) Comparison of 1-CDF(number of cells) (percent of simulations with more than N cells at a given age) for the 100,000 simulations and the model prediction for the same parameters. Dashed dark blue line: model prediction using simulated parameters. Light blue area: empirical probability of observing more than N cells at a given age for the simulated parameters (*μ*_1_ = 3.1, *b*_1_ = 9, *d*_1_ = 8.8, *μ*_2_ = 10^−5^, *b*_2_ = 9.2, and *d*_2_ = 8.8). (B-D) Empirical MCMC-derived 2D density of posterior distribution of parameters. Warmer color indicate parameter values which are more likely to have produced the data. Black dots indicate the simulated parameter values: *μ*_1_ = 3.1, *b*_1_ = 9, and *γ*_1_ = .2

We compare our model predictions regarding the distribution of the cell numbers in compartment A (expressed by the empirical cumulative distribution functions, CDFs) to the simulated data and find that they are indistinguishable (Fig 3A). Maximum likelihood estimation via Nedler-Mead optimization demonstrates that we can recapture the parameters used in the simulation of the data. This is seen further in the posterior parameter distributions generated via MCMC; the simulated model parameter values are well-placed within the 2D posterior densities from the MCMC chain across the parameter space (Fig 3B–3D), indicating good agreement of approximation and simulation.

**Fig 4:**
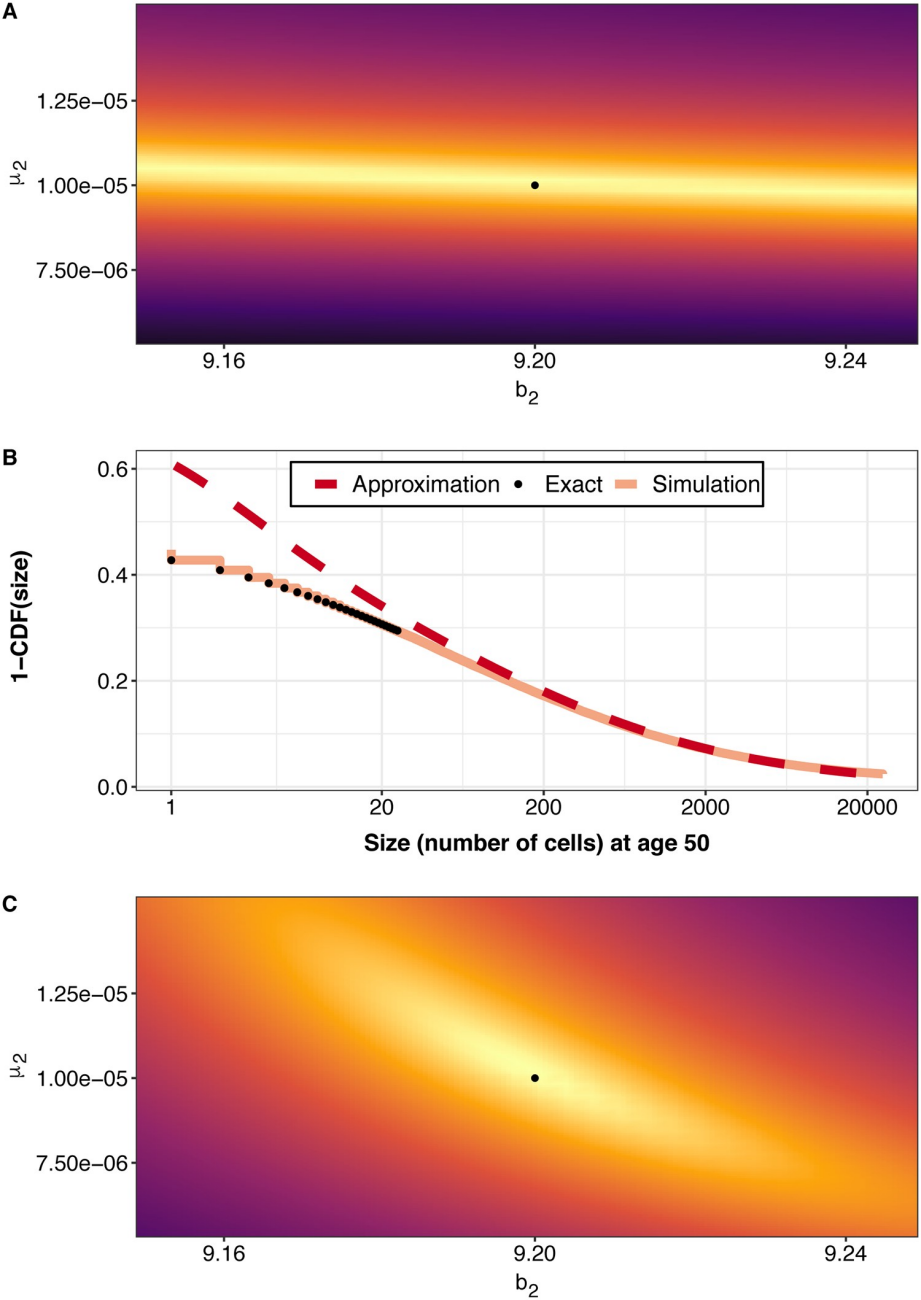
Agreement of simulation and mathematical model for compartment *M*. (A) Prevalence-only likelihood landscape which takes into account extinction of compartment *M*. Warm colors indicate parameter values which are more likely to have produced the data, variation in warm-ridgeline band is an artifact of grid choice. (B) Comparison of empirical 1-CDF(number of cells) (Percent of simulations with more than N cells) at age 50 for the simulated data and our new approximation. Separation at low sizes demonstrates that our approximation is most accurate at large ages. Black dots are exact calculation of probabilities taking the derivative of the probability generating function. (C) Likelihood landscape around our utilized parameters using our new approximation and the complete empirical size distribution of the simulated data. Warmer regions indicate parameter values which are more likely to have produced the data. The black dots indicate our biologically simulated parameters: *μ*_1_ = 10^−5^, and *b*_1_ = 9.2.

Similar to compartment *A*, we are able to fully recapture our parameters for compartment *M*. Without our approximation for the density of the compartment *M*, we would be forced to evaluate the parameter space with a likelihood which only takes into account prevalence data, i.e., carcinoma yes/no, (S1 Appendix Eq. 46) and would be unable to identify the best parameter combination of *μ*_2_ and *b*_2_ (Fig 4A). With our approach, however, we can closely approximate the CDF of the distribution of sizes in compartment *M* at a given age (Fig 4B). While our approximation only agrees with exact calculations for large sizes, in practice, detectable cancers and adenomas will be always within this large-size regime (>100 cells). This allows us to take advantage of more data, namely, the actual size information of a particular growth, and leads to successful parameter inference and the identification of the parameters used to simulate the data (Fig 4C).

**Fig 5:**
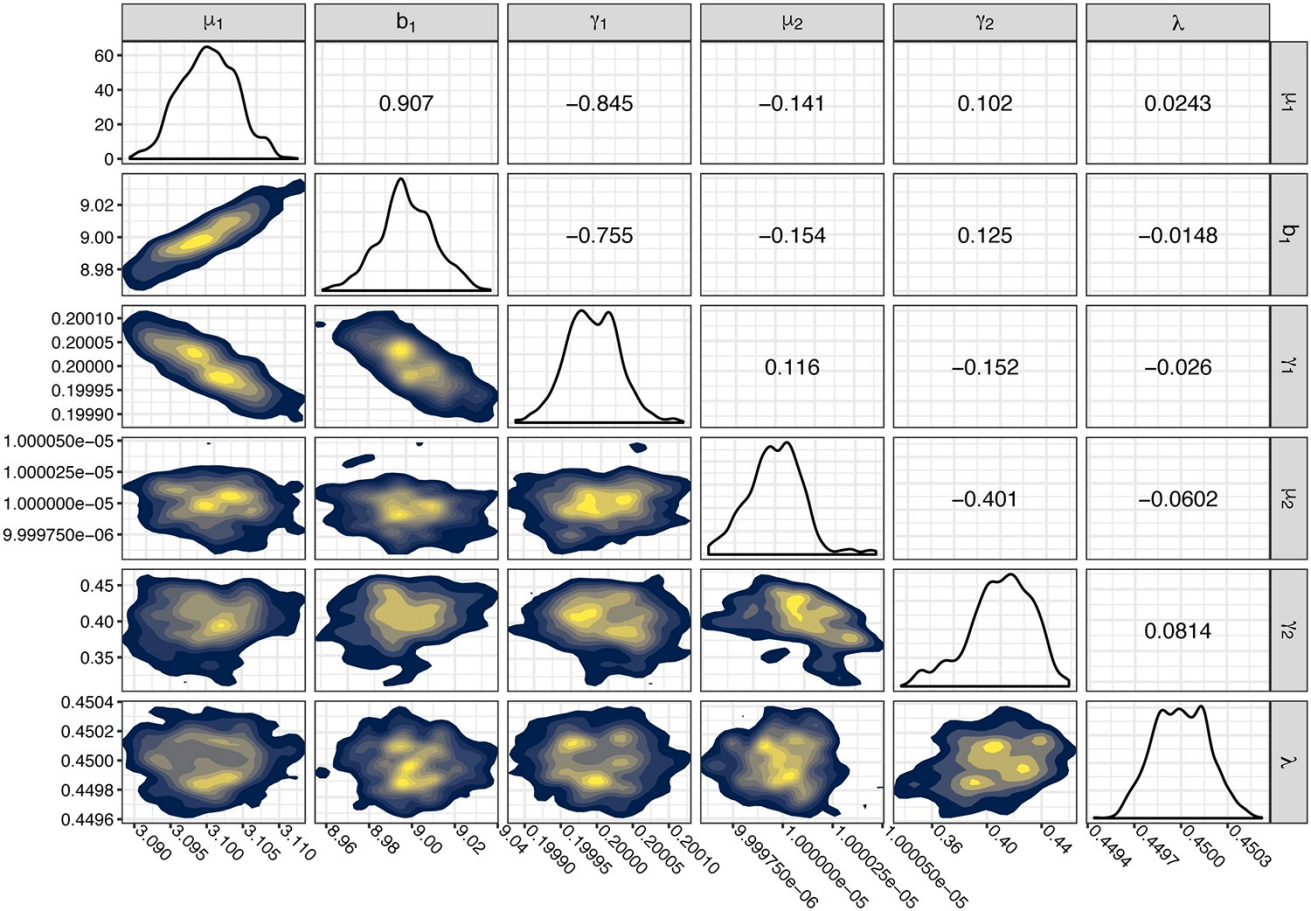
Agreement of simulation and mathematical model for compartments *A* and *M*. We performed 10,000 steps of adaptive MCMC on the simulated data and present the posterior distributions of the chain. Parameters used in the simulated data are: *μ*_1_ = 3.1, *b*_1_ = 9, *γ*_1_ = .2, *μ*_2_ = .00001, *γ*_2_ = .4, λ = .4. All parameters besides λ have the units per cell per year. λ is a population proportion. Upper right triangle: Pairwise parameter correlation. Diagonal: Univariate density of posterior parameter distribution. Lower left triangle: 2D posterior density distributions for pairs of parameters. Warmer colours indicate parameter values which are more likely to have produced the data.

After demonstrating that we can recapture parameters for each compartment individually, we perform adaptive MCMC to explore the parameter space of both compartments simultaneously for the simualated data. For this full model parameter inference, we add a number of zero-size observations to the simulated data, to model adenoma-resistant individuals. These imputed zeros make up λ = 40% of our observations. We find that the simulated model parameters are very close to the model parameters that have the highest likelihood (Fig 5).

**Fig 6:**
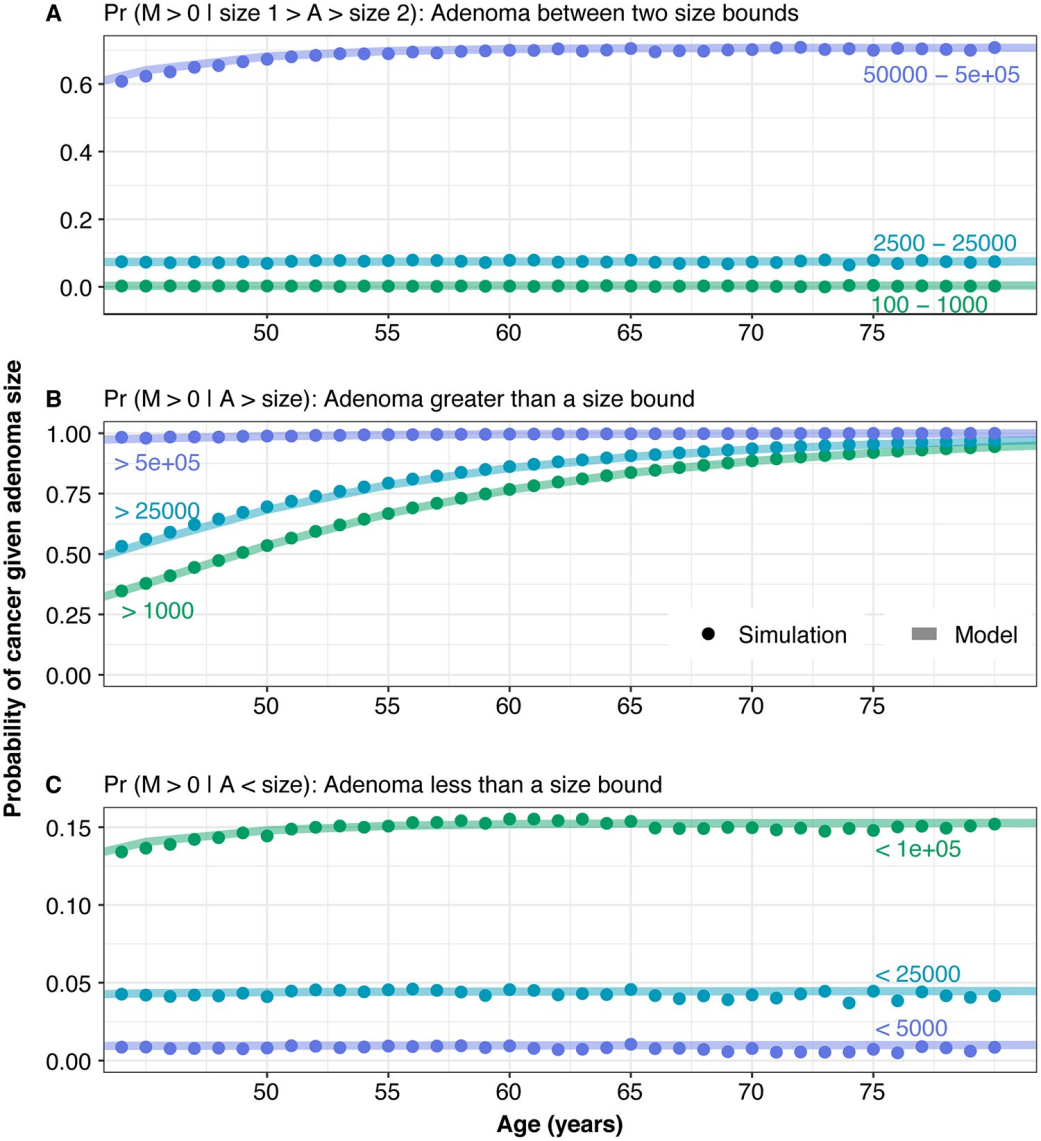
Agreement of simulation and mathematical derivations regarding the conditional probability of cancer given number of adenoma cells. We compare the empirical probability of cancer given three size ranges (points), as derived from the simulations and compare this to our model derived values (lines). (A) Probability of cancer given compartment *A* has 100-1000, 2500-25000, or 50000-500000 cells. (B) Probability of cancer given compartment *A* has more than 1000, 25000 or 500000 cells. (C) Probability of cancer given compartment *A* has fewer than 5000, 25000 or 100000 cells.

We are ultimately interested in calculating the probability of cancer depending upon the size of the adenoma compartment *A*. We compute the probability of non-extinction in compartment *M* given a certain number of cells in compartment *A* (S1 Appendix Eq. 53). Given successful parameter inference, this quantity represents the probability that an individual with an adenomatous polyp of a given size also has cancer. We compare model-predicted conditional probabilities of cancer cells in compartment *M* given that compartment- *A* size has a lower bound, an upper bound, or is between two bounds with the empirical probabilities from the simulated data and find good agreement (Fig 6).

### Parameter inference on real data

**Fig 7:**
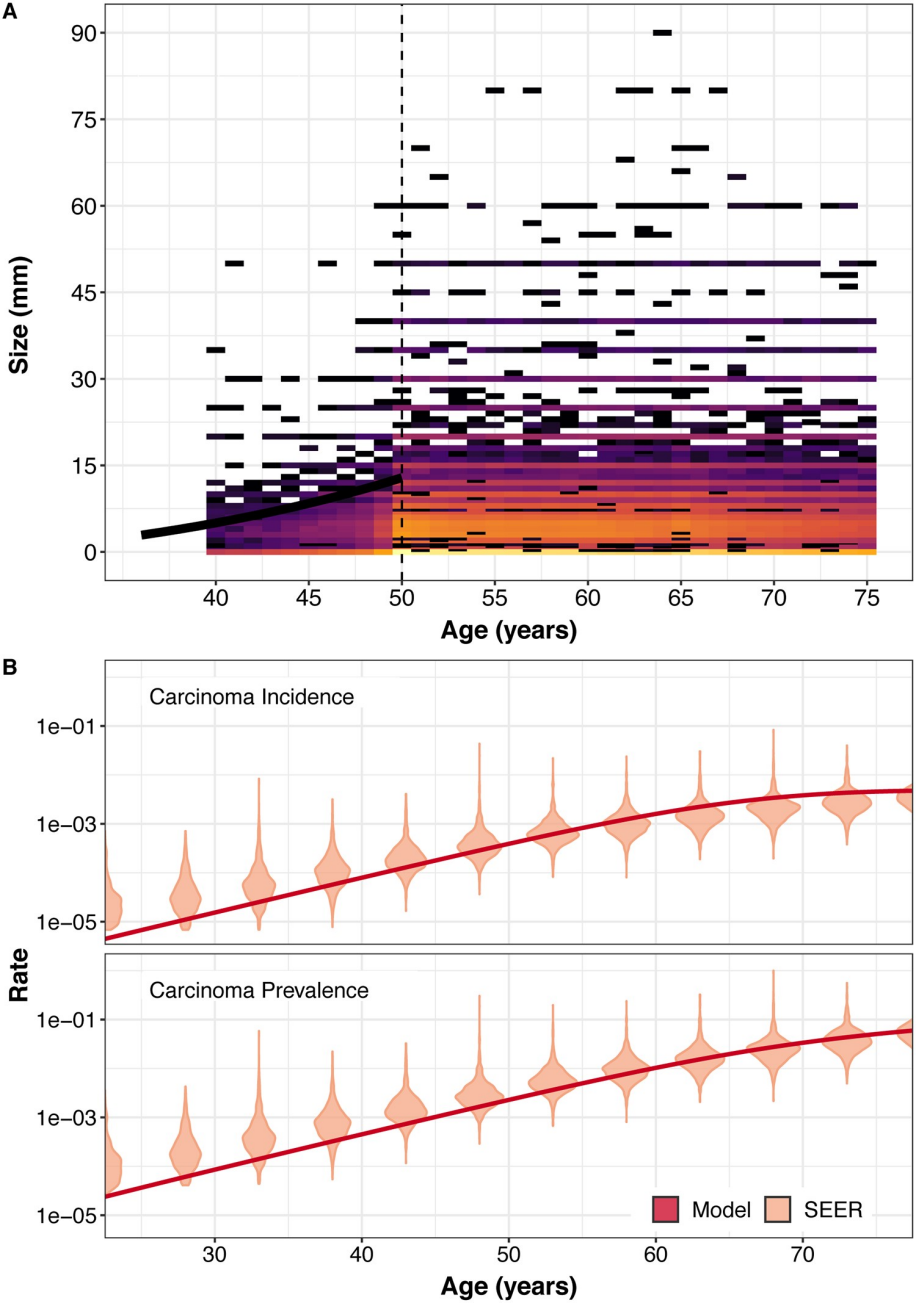
Model prediction of real-world adenoma size and rates of carcinoma. (A) Average adenoma size in mm. Black line: Model predicted average adenoma size layered on top of binned count data for the CORI data. Colored bins: Colored bins: Number of individuals in the CORI data set with a reported adenoma of a given size. Dashed line: Beyond age 50 we do not see an age-dependent increase in average adenoma size and this data was excluded for our calculations. (B) Cancer prevalence and incidence rates. Red lines: Model-predicted cancer incidence and prevalence rates for given ages. Violin plots: Density of estimated rates for 5-year age-bins as derived from the SEER data.

We now want to infer model parameters that allow us to reflect the true prevalence of colorectal adenoma and cancers. Therefore, we fit the complete model on the binned adenoma size data from the CORI endoscopic database and binned cancer size data from SEER registry. The model was fit using a combined (composite) likelihood for compartments *A* and *M* allowing for an sub-population of individuals of size 55% which will neither develop cancer or adenoma (Eq 13 and Fig 7).

**Fig 8:**
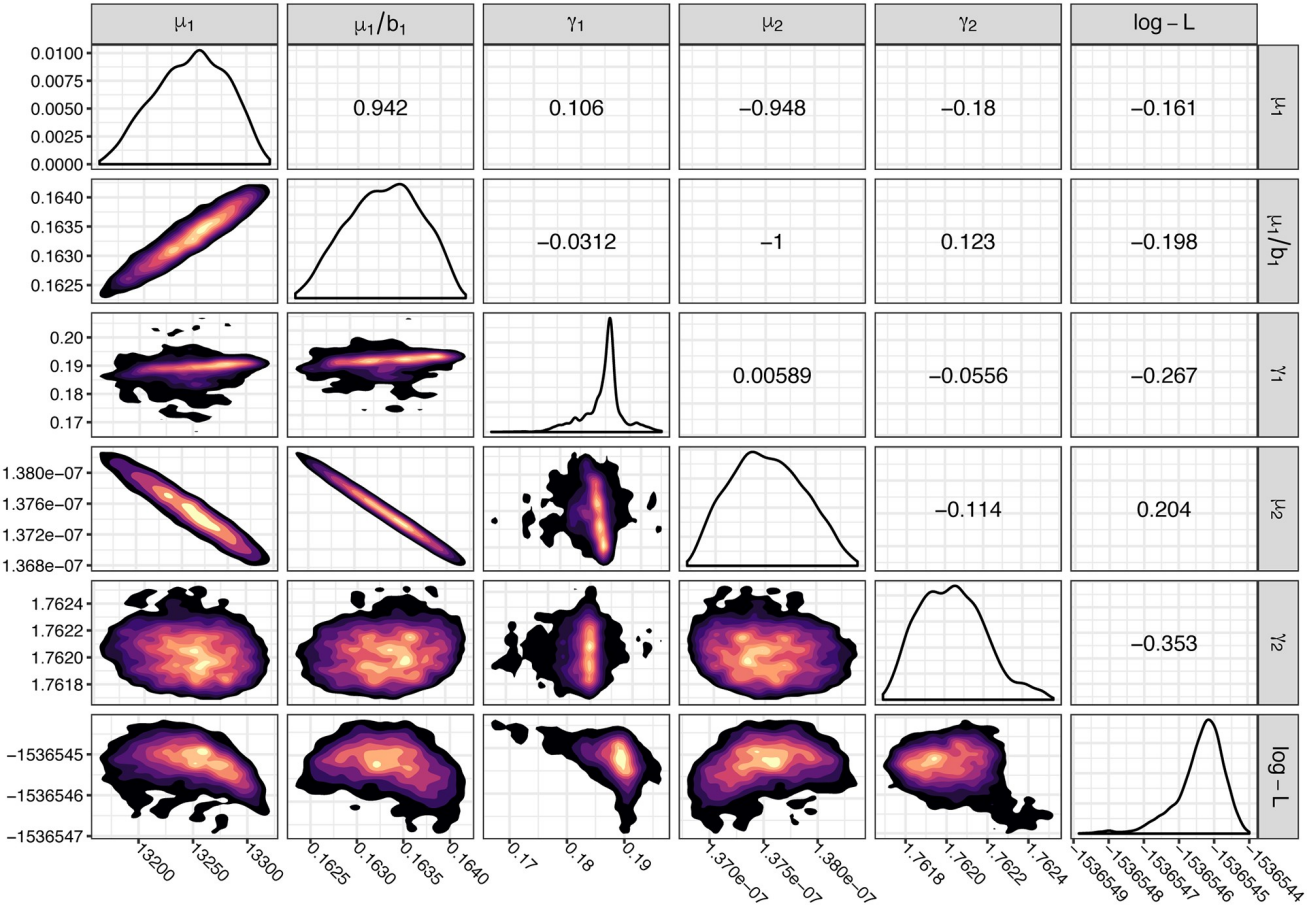
Parameter distributions and correlations for the model fit to CORI and SEER data. We performed 10,000 steps of adaptive MCMC on the parameter space, evaluated on the CORI and SEER data and present the posterior distributions of the chain. All parameters have the units per cell per year. Upper right triangle: Pairwise parameter correlation. Diagonal: Univariate density of posterior parameter distribution. Lower left triangle: 2D posterior density distributions for pairs of parameters. Warmer colours indicate parameter values which are more likely to have produced the data.

The inferred parameters of our model can be interpreted in biological terms: the best-fit immigration rate into compartment *A* (adenoma initiation), *μ*_1_, was found to be 13,200 cells per year. The best adenoma net-growth rate *γ*_1_ = *b*_1_ − *d*_1_ was found to be 0.165 (an increase of 16.5% per year) per cell per year. The model was able to recapture the growth in average size seen in the CORI data up to age 50 (Fig 7A). For transition from adenoma to cancer, we found *μ*_2_ to be 1.38 × 10^−7^ per cell per year. The net-growth rate of compartment *M*, *γ*_2_, was found to be 1.76 (an increase of 176% per year) per cell per year. We found good visual correspondence between the incidence and prevalence rates from the SEER data and incidence and prevalence predicted by our model (Fig 7B). With our likelihood approach we are able to perform adaptive MCMC run across the parameter space, giving us the posterior distribution of the parameters of our model, given the data (Fig 8).

### Probability of CRC presence given detection of adenoma

**Fig 9:**
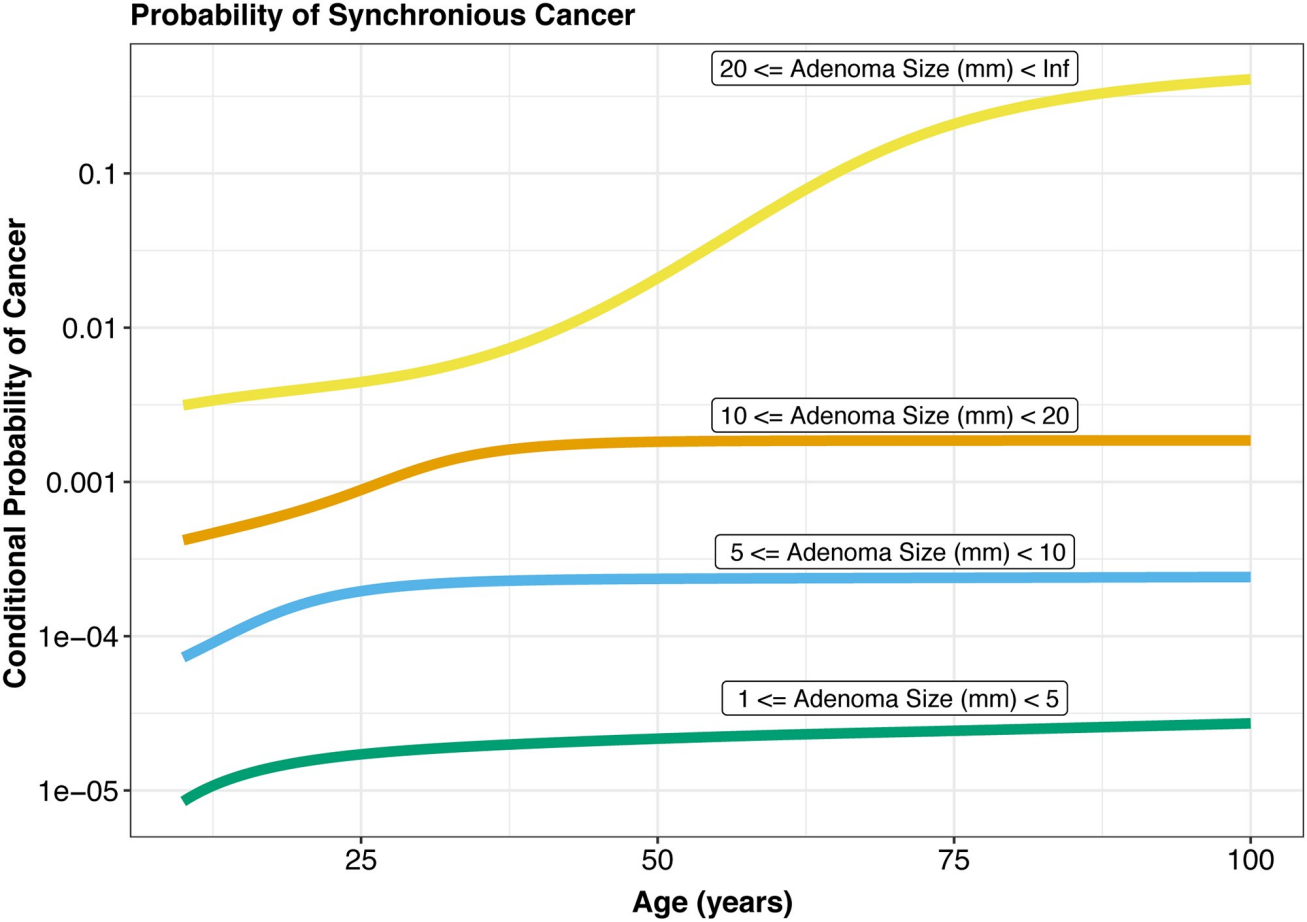
Conditional probability of cancer given adenoma size for CORI and SEER data-derived parameters. Predicted probability of cancer given adenoma prevalence of a particular size. Parameters used are inferred from the two-type branching process model fit upon the CORI and SEER data. Labels indicate size of adenoma growth. Y-axis denotes the probability of cancer presence given adenoma size.

With our inferred model parameters across the two data sources we can now compute the synchronous probability of colorectal cancer given the presence of an adenoma of a particular size. We compute these probabilities of cancer for individuals with adenomas between four ranges: <5 mm adenoma, between 5 and <10 mm adenoma, between 10 and <20 mm adenoma, and an adenoma equal or greater than 20 mm in size. The sizes here correspond to the endoscopist-estimated largest dimension in mm. We find that for patients over the age of 50, the probability that an individual would have cancer given an observed adenoma between 1 and <5 mm is 1/42000. For larger adenoma size ranges the probability of cancer increases, and we observe probabilities of 1/3900 for patients with adenomas between 5 and <10 mm in size and 1/500 for patients with adenomas between 10 mm and <20 mm in size. For patients with adenomas larger than 20 mm, our model predicts cancer rates of 1/40 for 50 year-olds, and 1/6 for 70 year-olds (Fig 9). An implementation of our model for prediction of carcinoma in an adenoma for patient ages between 30 to 70 years and adenoma sizes from 0 to 30 mm, is freely available as an R/shiny web-app at https://ccrc-eth.shinyapps.io/CCRC/.

## Discussion

We have presented a model of the dynamics of colorectal cancer development using a two-type branching process. The fitting of this model to the CORI and SEER data is enabled through our new approximation to the size distribution of the carcinoma compartment at time *t*, *M*(*t*). We present new estimates of the rates defining average risk (spontaneous) colorectal carcinoma development. Our estimated parameters indicate fast transition from adenoma to carcinoma, and a similarly fast tumor volume doubling time.

Our new approximation to the two-type branching process with immigration enables the computation of the size density of the carcinoma compartment (compartment *M*). Previous efforts to solve this system have stopped short, and in these studies only the probability generating function of compartment *M* was provided [37]. In such a model, numerical computation of the probability of a large number of cells in compartment *M* would be practically impossible. With our approximation to this distribution, these probabilities can now be efficiently computed.

Efficient calculation of the adenoma compartment, in turn, enables the application of the two-type branching process model to epidemiological data. Parameters of our model could thus be learned using real-world data regarding adenoma prevalence and size from the CORI database and carcinoma incidence from the SEER database. With our approach and the access it grants to the computation of a size-based likelihood, enabling us to search the parameter space and can describe the space with MCMC-based posterior density estimates.

For the initiation of adenoma cells, we find that the immigration rate into compartment *A* is 13,200 cells per year. As described in the methods, our prior expectation was centered on a rate of 3100 cells per year. While the two-stage branching process model has been shown to incapable of explaining the two-hit mutational process of adenoma initiation, we draw parallels to that process to educate our prior expectations [23]. As our prior expectation is based on a composite of biologically feasible somatic mutation rates, the number of active stem cells, colonic crypt number, and number of base pairs of the genetic regions which–when mutated–could lead to cancer, the difference here could be explained by variations in any or all of these values. The use of the two-stage branching process model, while it provides good opportunity to leverage adenoma and cancer size data to gain new insights, constitutes a trade-off when it comes to the interpretation of adenoma initiation.

The inferred net growth of an adenoma of 16.5% per year is consistent with previous estimations of adenoma growth in general [23, 46] and suggests that an adenomatous polyp would take, on average, 4.5 years to double in volume, reinforcing evidence that such polyps develop slowly over many years [26, 49]. This corresponds to an average of 21.9 years to grow from 3mm to 10mm in endoscopist-reported largest dimension. The subsequent growth from 10mm to 30mm would, on average, take another 20.0 years.

For compartment *M*, we find that the mutation rate from adenoma into cancer is 1.38 × 10^−7^ per year per adenoma cell, several orders of magnitude faster than the somatic mutation rate of a normal colonic stem cell, but similar to the average of the male and female rates estimated in previous modeling studies [22, 23, 50]. This average rate may be misleading, however, as it has been shown that potentially only 1-10% of adenoma cells are capable of malignant transition [25]. This suggests that the true rate among those cells may be up to two orders of magnitude faster. Similar to this fast transition rate, we estimate the net growth rate for an initiated cancer to be 176% per year. This rate would correspond to growth from a single cell to a size larger than 2.5mm in less than 7.35 years, or a doubling time of 250 days. With these numbers we could simulate the two-type branching process to make time-based predictions about the probability of a cancer of a certain size in the future given a current adenoma size. However, these simulations would be very computationally burdensome and without additional experimental or epidemiological validation, these predictions should not be used for medical decision-making.

Considering both compartments jointly, the parameters can be used to calculate the average sojourn time of an adenoma growth, i.e., the time it takes for a single adenoma cell to grow and produce its first cancer cell, conditioned on non-extinction. Our calculated value of 49.2 years suggests an extremely slow transition period and is consistent with values found with the application of other models [23]. Recently published work by Luebeck et al. supports even longer pre-malignant periods, as well as demonstrating timing differences between cancer development in the proximal and distal colon and rectum [51].

Conditioning on an adenoma finding of size between 1 and 5mm, our inferred model parameters predict a cancer rate very close to 0.00008, a bit below a cancer rate of 1/3744 as found in the literature [52]. At larger adenoma sizes, we predict rates more similar to those found previously. For findings of size between 5 to <10 mm (0.0013 vs. 2/1198 = 0.0016), and 10 to <20 mm (0.01 vs. 16/963 = 0.016) the predictions are very close to those seen in observational studies [52].

In our study, fitting of parameters describing the adenoma compartment were dependent on the CORI database, a registry of endoscopic procedures [38]. We filtered data to include only individuals at average risk with a first screening procedure. As seen recently, already with individuals 50 years old we note that the average recorded size of adenomas is no longer associated with age [51]. While expected due to the limited growth space of the colon, this led us to focus on individuals younger than 50 years of age where we still observed an age-size relationship of adenomas (Figure A in S1 Appendix). However, screening for most individuals is only recommended at or beyond 50 years of age [10, 11] and data from fewer individuals at average risk in the age group 40–49 were available. In addition, the data provided for patients under 50 years of age will be enriched with patients who have non-average risk characteristics, and the growth rates of these patients may be substantially different from the average-risk population.

The SEER data is a registry of cancer incidence and only detected cancers will be recorded [40]. In this way the database potentially misses many individuals who have cancer at a given age, but have not yet been detected. Additionally, our model likelihood requires counts for the number of prevalent cancer cases, while the SEER registry comprises incidence cases that have been subsequently removed from the cancer pool, effectively censoring their cancer sizes and prevalence. To cope with this we computed population prevalence from the SEER data and used this to correct our size data. Uncertainty in our correction may bias our results.

In the future the learned model parameters can be applied to simulations regarding the efficacy of colorectal cancer screening strategies. In practice, however, this is very costly due to the size of our model parameters. Other routes could be explored, for example tau-leaping [53], to simulate the model with the inferred parameters and directly assess further quantities of interest.

The two-type branching process model as applied to colorectal cancer in this paper could be used to simulate any process with phenomenological similarity. In particular, one could apply our approach to any cancer with defined and quantifiable precursor stages such as Barrett’s carcinoma of the esophagus [54], anal carcinoma with condyloma precursors [55] and gastric carcinoma derived from gastric metaplasia and dysplasia [56]. However, for cancers other than colorectal cancer, these two stages are less defined or measurable, and the utility of our model is limited.

Our work has several strengths: First, this work has extended the utility of the two-type branching process model and provides the ability to perform maximum likelihood estimation and broad parameter fitting. Second, we related the model to real-world epidemiological data regarding adenoma prevalence and characteristics as well as carcinoma incidence. And third, new posterior density estimates from the MCMC provide a strong estimate for the realm of plausible parameters which could generate adenoma and carcinoma sizes seen in real-world data.

We also note a number of limitations of our work: First, the two-stage branching process model may not fully capture the initiation trends of the adenoma compartment, due to the biological two-hit mutational process underlying this initiation. We have, however, solved the model to allow for likelihood-based parameter inference and have found that our inferred parameters fit quite well with what has been previously found. Second, our approximation only provides reliable estimate for large numbers of cells in compartment *M* and size probabilities for less than 50–100 cells will be increasingly inaccurate. These numbers of cells, however, are far below the standard detection limit of colonoscopy, so this limitation has no real practical consequence. Third, the generating function for compartment *M* and hence its approximation involve combinations of hypergeometric functions, the implementation of which can be challenging. Fourth, key aspects of the natural history of CRC such as the joint probability of compartments *A* and *M* or the presence of multiple adenomas or synchronous carcinomas have not been calculated in our approach. With the currently available mathematical tools we are able to predict current cancer existence given current adenoma size, however, time-based predictions of future cancer occurrence given current adenoma size, or the prediction of cancer size given adenoma size, could only be addressed by stochastic simulation of the system with our currently inferred parameters, which would be computationally demanding. Multiple adenomas or carcinomas is not accounted for in the two-type branching process model. Fifth, for simplicity we fix death rates of adenoma and carcinoma cells to be equal and in doing so we assume that transition to cancer will exclusively effect the net growth rate *γ*_2_ through variation in compartment-*M* birth rate *b*_2_. Comparisons between compartments *A* and *M* are thus limited to those of net growth rates *γ*_1_ and *γ*_2_. However, mutations in carcinoma cells can affect both, growth and survival, which will not be adequately reflected by our model. In addition to this, we further restrict our model with the inclusion of time-constant parameters. While there are models, such as the Bellman-Harris process [57], which allow for time-dependent growth rates and could possibly fit the trends seen in cancer more closely, the solution of these more complex models to fit size data in a similar approach presented in the paper is an open challenge. Sixth, a resistance population parameter λ accounting for the fraction of individuals who never develop carcinoma was included to allow for some individual variability but this is also potentially problematic if, given a long enough lifetime, all individuals of a population will experience cancer. In the future, we imagine allowing birth and death rates to follow a distribution to account for uncertainty and variation in growth rates across the population. Moreover, throughout our models, we do not discern between patient subgroups such as sex, race, and colon location. In the future, our model could be adapted to these patient groups simply through multiple fittings, but the SEER database is limited due to the significant variation in level of reporting among patient subgroups. Also, differences in individual cancer risk are not accounted for by our modeling, and our model rather assumes that all parameters are shared across the population. However, it is likely that each person’s cells have a propensity for cancerous growth which varies due to a variety of causes such as individual genetic predisposition, immune system activity [58], microbiome [59], and lifestyle [60]. Finally, for parameter estimation of our model the CORI and the SEER database were used and biases in collection (see above) as well as inconsistencies in recording of data will also affect our results.

In summary, our work applies the two-type branching process model to colorectal cancer development and enables direct calculation of the size of the pool of cancer cells. This mathematical advancement allows for parameter estimation using data from large databases and thus allows for a more precise estimation of all transition rates including the transition from adenoma to carcinoma cells *μ*_2_. While previous models could only use binary prevalence but not size data, our approach enables us to fit model parameters to data on adenoma and carcinoma size, providing improved estimates of the rates of CRC development. Understanding the differences in these rates may be used to inform further discussions about the natural history of CRC, which will impact on utility and timing of screening guidelines.

## Supporting information

S1 Appendix Mathematical development and supplementary figures. (PDF)
