# Supplementary material for: Predicting colorectal cancer risk from adenoma detection via a two-type branching process model
Source: PLoS Comput Biol. 2020 Feb 5;16(2):e1007552. doi: 10.1371/journal.pcbi.1007552 (PMC7001909; doi:10.1371/journal.pcbi.1007552)
Supplement: S1 Appendix — (PDF) [file pcbi.1007552.s001.pdf]

S1 Appendix for "Predicting colorectal cancer risk from adenoma detection via a two-type branching process model"

Brian M. Lang, Jack Kuipers, Benjamin Misselwitz, and Niko Beerenwinkel

1 Mathematical Development

1.1 Compartment-A evolution

In this section we highlight how to compute the evolution of the probability of having a given number of cells in compartment A. This is a standard result, and included for completeness. We start with a birth-death process with immigration

$$\begin{aligned} \emptyset &\xrightarrow{\mu_1} A \\ A &\xrightarrow{b_1} A + A \\ A &\xrightarrow{d_1} \emptyset \end{aligned}$$

We encode the dynamics of the stochastic process in the probability generating function

$$G(s,t) = \sum_{n=0}^{\infty} s^n \mathrm{P}(A = n) \tag{1}$$

which evolves, due to the master equation, according to the following partial differential equation

$$G_t = [b_1 s(s-1) + d_1(1-s)]\, G_s + \mu_1(s-1)G \tag{2}$$

Writing this as

$$G_t - v \cdot G_s = \mu_1(s-1)G \tag{3}$$

and using the method of characteristics one has

$$G(s(0),t) = G(s(t),0)\mathrm{e}^{\mu_1 \int_0^t (s(t')-1)\mathrm{d}t'} \tag{4}$$

where $s(t)$ evolves under the ordinary differential equation $\dot{s} = v$. In our case this is

$$\dot{s} \;=\; b_1 s(s-1) + d_1(1-s) \tag{5}$$

If we start with no cells, then $G(s,0) = 1$ and

$$G(s(0),t) = \mathrm{e}^{\mu_1 \int_0^t (s(t')-1)\mathrm{d}t'} \tag{6}$$

Solving the differential equation

$$s(t) = 1 + \frac{\gamma_1(1-s(0))}{b_1(s(0)-1) - (b_1 s(0) - d_1)\mathrm{e}^{-\gamma_1 t}} \tag{7}$$

with $\gamma_1 = b_1 - d_1$. Then

$$\mu_1 \int_0^t (s(t')-1)\mathrm{d}t' = \frac{\mu_1}{b_1} \ln\left[\frac{\gamma_1}{(b_1 s(0) - d_1) - b_1(s(0)-1)\mathrm{e}^{\gamma_1 t}}\right] \tag{8}$$

giving

$$G(s,t) = \left[\frac{\gamma_1}{(b_1 s - d_1) - b_1(s-1)\mathrm{e}^{\gamma_1 t}}\right]^{\frac{\mu_1}{b_1}} \tag{9}$$

1.1.1 Probability distribution of $A(t)$.

Next we expand in powers of s to get the probability distribution over time. First we write the term in the bracket as

$$\frac{\gamma_1}{(b_1 e^{\gamma_1 t} - d_1) + b_1 s(1 - e^{\gamma_1 t})} = \frac{1-p}{1-ps} \tag{10}$$

with

$$p = \frac{b_1(e^{\gamma_1 t} - 1)}{(b_1 e^{\gamma_1 t} - d_1)} \tag{11}$$

The generating function, with $r = \frac{\mu_1}{b_1}$ is

$$G(s,t) = (1-p)^r \left[\frac{1}{1-ps}\right]^r \tag{12}$$

so the expansion is simply a negative binomial since

$$(1-ps)^{-r} = \sum_{k=0}^{\infty} \binom{r+k-1}{k} (ps)^k \tag{13}$$

For non-integer r the binomial coefficient is defined as

$$\binom{r+k-1}{k} = \frac{\Gamma(r+k)}{\Gamma(r)\Gamma(k+1)} \tag{14}$$

As mentioned in the main text (Eq. 5), the probability $P(A(t) = k)$ of having k individuals at time t in the first compartment is then

$$P(A(t) = k) = (1-p)^r \binom{r+k-1}{k} p^k \,, \qquad K \sim \mathrm{NB}\,(r, p) \tag{15}$$

with CDF

$$\Pr(A(t) \leq k) = 1 - \frac{\mathrm{B}\,(p; k+1, r)}{\mathrm{B}\,(k+1, r)} \tag{16}$$

in terms of the Beta function B. With this we can assess the likelihood of a patient having a size range of $[U_i^A, L_i^A]$ at time t_i^A

$$\mathcal{L}(\Theta^A \mid O_i^A) = \frac{\mathrm{B}\left(p; L_i^A + 1, r\right)}{\mathrm{B}\left(L_i^A + 1, r\right)} - \frac{\mathrm{B}\left(p; U_i^A + 1, r\right)}{\mathrm{B}\left(U_i^A + 1, r\right)} \tag{17}$$

As in the main text (Eq. 12), if we assume that immune individuals are distributed throughout the population with proportion λ we then have a mixture model which has likelihood:

$$\mathcal{L}(\Theta^A, \lambda \mid O_i^A) = \begin{cases} \lambda + (1-\lambda)\ \Pr(L_i^A \leq A(t) \leq U_i^A) & \text{if } 0 \in [L_i^A, U_i^A], \\ (1-\lambda)\ \Pr(L_i^A \leq A(t) \leq U_i^A) & \text{if } 0 \notin [L_i^A, U_i^A] \end{cases} \tag{18}$$

1.2 Compartment-M evolution

1.2.1 Compartment-M generating function.

In this section we detail the computation of the probability generating function of the number of cells in the second compartment. This is a minor modification of the result without immigration of [1], and is included to define the functions which form the basis of our inference.

We have a two-type branching process with immigration

$$\begin{array}{ccc} \emptyset & \xrightarrow{\mu_1} & A \\ A & \xrightarrow{b_1} & A+A \\ A & \xrightarrow{d_1} & \emptyset \\ A & \xrightarrow{\mu_2} & M \\ M & \xrightarrow{b_2} & M+M \\ M & \xrightarrow{d_2} & \emptyset \end{array}$$

for which the probability generating function satisfies

$$\begin{aligned} G_t &= [b_1 s_1(s_1-1) + \mu_2(s_2-s_1) + d_1(1-s_1)]\, G_1 \\ &\quad + [b_2 s_2(s_2-1) + d_2(1-s_2)]\, G_2 + \mu(s_1-1)G \end{aligned} \tag{19}$$

where $\boldsymbol{s}(t)$ evolves under the ordinary differential equation $\dot{\boldsymbol{s}} = \boldsymbol{v}$. In our case this is

$$\begin{aligned} \dot{s}_1 &= b_1 s_1(s_1-1) + \mu_2(s_2-s_1) + d_1(1-s_1) \\ \dot{s}_2 &= b_2 s_2(s_2-1) + d_2(1-s_2) \end{aligned} \tag{20}$$

which we need to solve for $\boldsymbol{s}$ since when we start with no cells, $G(\boldsymbol{s},0)=1$ and

$$G(\boldsymbol{s}(0),t) = \mathrm{e}^{\mu_1 \int_0^t (s_1(t')-1)\mathrm{d}t'} \tag{21}$$

The second line of Eq. (20) can be solved easily

$$\int_{s_2(0)}^{s_2(t)} \frac{\mathrm{d}\tau}{(1-\tau)(d_2-b_2\tau)} = \int_0^t \mathrm{d}t' \tag{22}$$

since

$$\frac{d_2-b_2}{(1-\tau)(d_2-b_2\tau)} = \frac{b_2}{b_2\tau-d_2} - \frac{1}{\tau-1} \tag{23}$$

so we have

$$[\ln(b_2\tau-d_2) - \ln(\tau-1)]_{s_2(0)}^{s_2(t)} = -\gamma_2 t \tag{24}$$

in terms of the growth rate $\gamma_2 = b_2 - d_2$ of the second compartment. The solution for s_2 follows as

$$s_2(t) = \frac{d_2(s_2(0)-1) - (b_2 s_2(0) - d_2)\mathrm{e}^{-\gamma_2 t}}{b_2(s_2(0)-1) - (b_2 s_2(0)-d_2)\mathrm{e}^{-\gamma_2 t}} \tag{25}$$

leading to

$$1 - s_2(t) = \frac{\gamma_2}{b_2(1-z)}\,, \qquad z = \frac{(b_2 s_2(0)-d_2)}{b_2(s_2(0)-1)}\mathrm{e}^{-\gamma_2 t} = \left[1 + \frac{\gamma_2}{b_2(s_2(0)-1)}\right]\mathrm{e}^{-\gamma_2 t} \tag{26}$$

Following [1] we define $x = 1 - s_1$ to arrive at the differential equation

$$\dot{x} = -b_1 x^2 + \gamma_1 x + \frac{\mu_2\gamma_2}{b_2(1-z)} \tag{27}$$

with $\gamma_1 = b_1 - d_1 - \mu_2$ the growth rate of the first compartment. We rescale $x = \frac{X}{b_1}$ to remove the first coefficient

$$\dot{X} = -X^2 + \gamma_1 X + \frac{\mu_2 b_1 \gamma_2}{b_2(1-z)} \tag{28}$$

and put it directly in the form in [1]. First they make the substitution

$$X = \frac{\mathrm{d}}{\mathrm{d}t} \ln Z \tag{29}$$

transforming to the differential equation

$$\ddot{Z} = \gamma \dot{Z} + \frac{\mu_2 b_1 \gamma_2}{b_2(1-z)} Z \tag{30}$$

The solution for Z can then be taken directly from [1] though we need to keep track of the fact that we divide X by b_1 which also affects κ which becomes

$$\kappa = \frac{b_1(s_1 - 1) - \omega}{\gamma_2 z_0} \tag{31}$$

with

$$\omega = -\frac{\gamma_1}{2} + \sqrt{\frac{\gamma_1^2}{4} + \frac{\mu_2 b_1 \gamma_2}{b_2}} \tag{32}$$

The solution for Z is then

$$Z(t) = z^{\frac{\omega}{\gamma_2}} \Phi(z) \tag{33}$$

with

$$\Phi(z) = F_1(z) + C z^{1-c} F_2(z) \tag{34}$$

with

$$F_1(z) = {}_2F_1(a, b; c; z)\,, \qquad F_2(z) = {}_2F_1(-a, -b; 2-c; z) \tag{35}$$

in terms of the hypergeometric function with parameters

$$a = \frac{\omega}{\gamma_2}\,, \qquad b = \frac{\omega + \gamma_1}{\gamma_2}\,, \qquad c = 1 + a + b \tag{36}$$

and

$$C = \frac{\kappa F_1(z_0) - F_3(z_0)}{(1 - c - \kappa z_0) F_2(z_0) + z_0 F_4(z_0)} \tag{37}$$

with

$$F_3(z) = {}_2F_1(1+a, 1+b; 1+c; z)\frac{ab}{c}\,, \qquad F_4(z) = {}_2F_1(1-a, 1-b; 3-c; z)\frac{ab}{(2-c)} \tag{38}$$

For our generating function with no starting cells we then just need to compute

$$G(\boldsymbol{s}, t) = \mathrm{e}^{\mu_1 \int_0^t (s_1(t') - 1)\mathrm{d}t'} = \mathrm{e}^{-r \int_0^t X \mathrm{d}t'} = \mathrm{e}^{-r[\ln Z]_0^t} = \left(\frac{Z(0)}{Z(t)}\right)^r \tag{39}$$

$$G(\boldsymbol{s}, t) = \left(\frac{F_1(z_0) + C z_0 F_2(z_0)}{F_1(z) + C z \mathrm{e}^{\gamma_2 c t} F_2(z)} \mathrm{e}^{\omega t}\right)^r \tag{40}$$

where we recall

$$z_0 = \left[1 + \frac{\gamma_2}{b_2(s_2 - 1)}\right]\,, \qquad z = z_0 \mathrm{e}^{-\gamma_2 t} \tag{41}$$

For the probability generating function only of cells in the second compartment, we can marginalize out the first compartment by setting $s_1 = 1$ so that κ simplifies to

$$\kappa = -\frac{\omega}{\gamma_2 z_0} \tag{42}$$

and we define

$$G(s, t) = G(\boldsymbol{s}, t)|_{s_1=1, s_2=s} \tag{43}$$

The approximation of the CDF of compartment M at time t starting from Eq. 40 is derived in the main text (Eq. 10).

1.2.2 Probability of no cells in the compartment M.

To compute the probability of having no cells in compartment M, we simply set $s_2 = 0$ so that

$$z_0 = \frac{d_2}{b_2} \qquad z = z_0 \mathrm{e}^{-\gamma_2 t}\,, \qquad \kappa = -\frac{\omega b_2}{\gamma_2 d_2} \tag{44}$$

The probability is therefore

$$P(M(t) = 0) = \left(\frac{F_1(z_0) + C z_0 F_2(z_0)}{F_1(z) + C z \mathrm{e}^{\gamma_2 c t} F_2(z)} \mathrm{e}^{\omega t} \right)^r \tag{45}$$

We can then describe the number of incident cases at age t, as $I(t)$, and size of the at-risk population at age t, as $R(t)$, to construct the likelihood of the parameters, Θ, given the data $O^I(t) = (I(t), R(t))$ at age t.

$$\mathcal{L}_{M(t)=0}(\Theta | O^I(t)) = \binom{R(t)}{I(t)} P(M(t) = 0|\Theta, t)^{I(t)} (1 - P(M(t) = 0|\Theta, t))^{(R(t)-I(t))} \tag{46}$$

This likelihood is used in the main text (Fig. 4) and represents the best-case likelihood prior to our approximation for the size distribution of $M(t)$

1.2.3 Probability of no cells in compartment M conditioned on compartment A.

$$G(s_1, M(t) = 0) = \left(\frac{F_1(z_0) + C z_0 F_2(z_0)}{F_1(z) + C z \mathrm{e}^{\gamma_2 c t} F_2(z)} \mathrm{e}^{\omega t} \right)^r \tag{47}$$

which now we want to expand in powers of s_1 through the dependence in κ. Since κ is linear in s_1 we can write the generating function in the format

$$G(s_1, M(t) = 0) = \left(\frac{J}{K - L s_1} \mathrm{e}^{\omega t} \right)^r \tag{48}$$

where the s_1 in the numerator cancels while we have formulae for the remaining coefficients:

$$J = \gamma_2 z_0 z^c \left[(c-1) F_1(z_0) F_2(z_0) - z_0 F_1(z_0) F_4(z_0) + z_0 F_3(z_0) F_2(z_0) \right] \tag{49}$$

$$\begin{aligned} K \;=\; & z_0 z^c F_1(z) \left[(\gamma_2 (c-1) - b_1 - \omega) F_2(z_0) - \gamma_2 z_0 F_4(z_0) \right] \\ & z_0^c z F_2(z) \left[(b_1 + \omega) F_1(z_0) + \gamma_2 z_0 F_3(z_0) \right] \end{aligned} \tag{50}$$

$$L = b_1 \left[z_0^c z F_1(z_0) F_2(z) - z_0 z^c F_1(z) F_2(z_0) \right] \tag{51}$$

The conditional distribution of compartment A given that there are no cells in compartment M is then just re-normalising by $P(M(t) = 0)$ for which we set $s_1 = 1$ to get

$$G(s_1 \mid M(t) = 0) = \left(\frac{K - L}{K - L s_1} \right)^r \tag{52}$$

which is the PGF of a negative binomial distribution with $p = \frac{L}{K}$ and $r = \frac{\mu_1}{b_1}$. With this result we can use Bayes' theorem to obtain the conditional probability of no cells in compartment M conditioned on the number of cells k in compartment A as follows:

$$P(M(t) = 0 \mid A(t) = k) = \frac{P(A(t) = k \mid M(t) = 0) P(M(t) = 0)}{P(A(t) = k)}. \tag{53}$$

This is then used to provide predictions of the existence of cancer given detected adenoma size in the main text (Figs. 6 and 9).

2 Number of cells in an observation

The size data provided in the CORI and SEER databases correspond to endoscopist-reported size in mm of the largest dimension of the finding. Assuming a half-ellipsoid shape and 10^8 cells per cm^3 of volume we convert this to a number of cells with the formula

$$\text{Cell number} = 10^5 \frac{4\pi}{3} \left(\frac{\text{size}}{2}\right)^3 \frac{1}{2}. \tag{54}$$

Each dimension of the ellipsoid is taken to be $\frac{\text{size}}{2}$ mm.

3 Figures

Figure A: **Illustration of CORI and SEER data.**
(A) Complete millimeter-binned CORI data, color indicates number of observations in a particular bin. Opaque bins greater than 49.5 years and black dashed line indicate data omitted in the fitting of compartment A. (B) Incidence rates of colorectal cancer as computed from the SEER registry. Colored lines indicate consistent registry, gender, birth year, and race. Opaque lines and black dashed lines indicate region of SEER data, below age 40 and above age 60, not utilized in the fitting of compartment M.

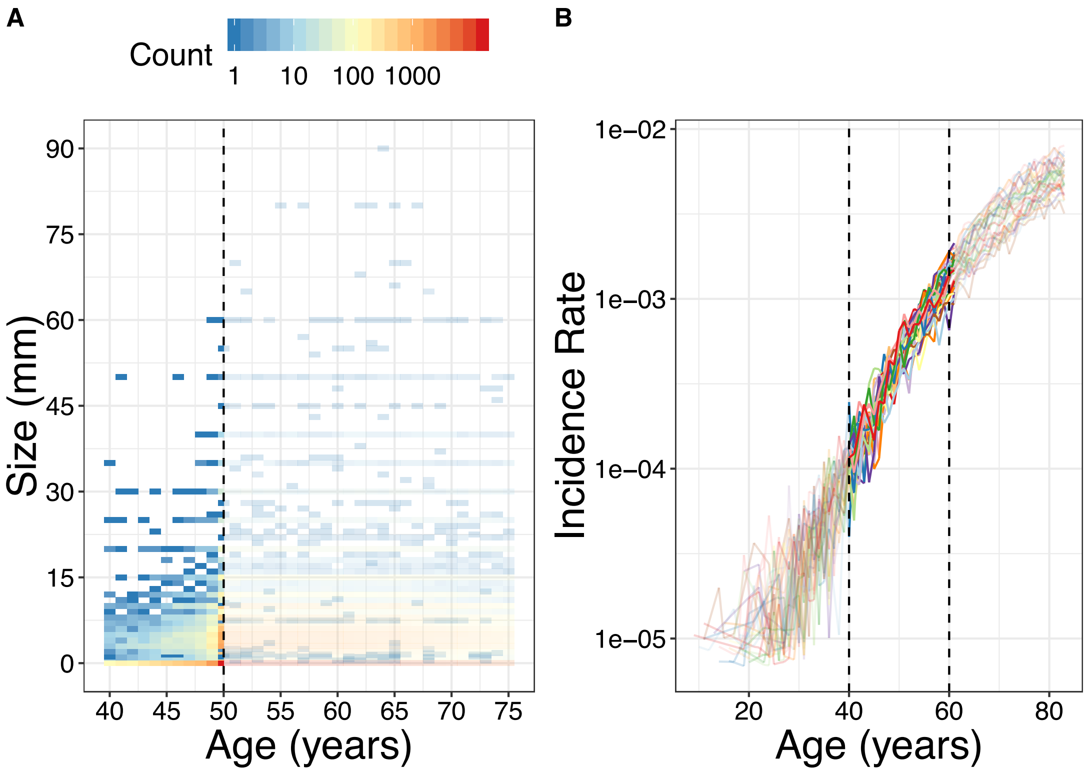

Figure B: **Illustration of pseudo population rates.**
Light pink (top bar) is the proportion of the population which are at-risk at a given age, $\hat{\mathrm{R}}(t)$. Dark pink (mid-bar) is the proportion of the population which are incident cases at a given age, $\hat{\mathrm{I}}(t)$. Middle pink (bottom bar) is the proportion of the population which have already been diagnosed with cancer by age t, $\hat{\mathrm{P}}(t)$.

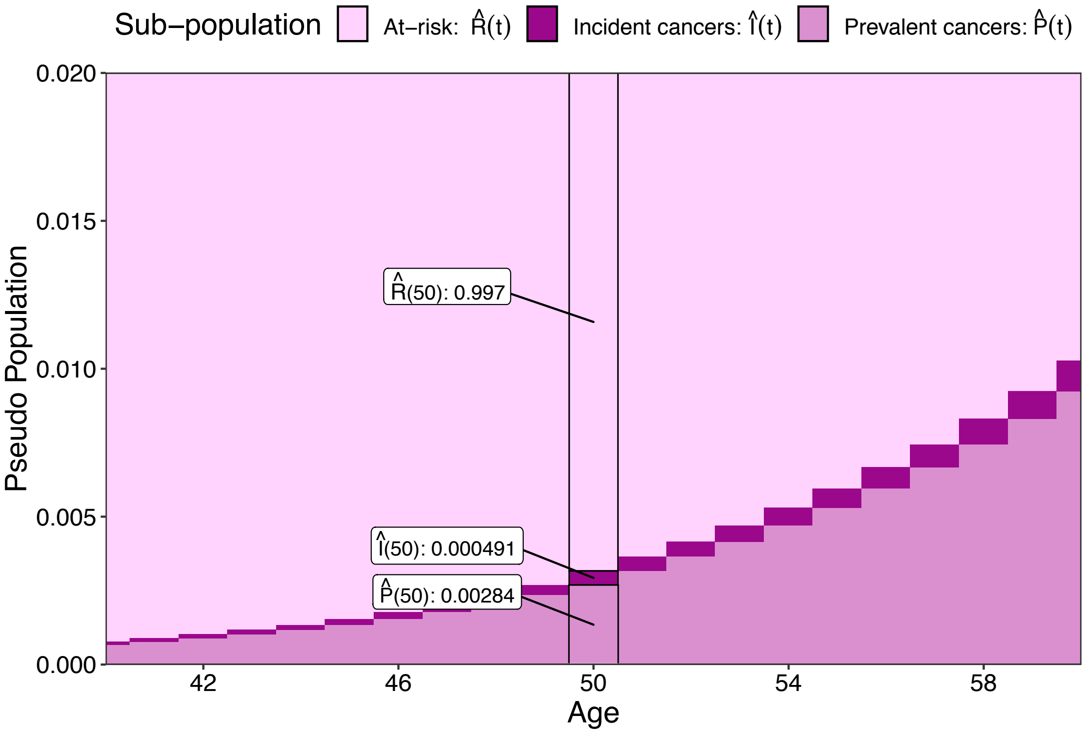

Figure C: **Age vs. Size in mm.**
Color indicates number of observations for each age-size pair. We see rapidly increasing average size until age 50, when screening begins, then the size distribution seems to stay constant.

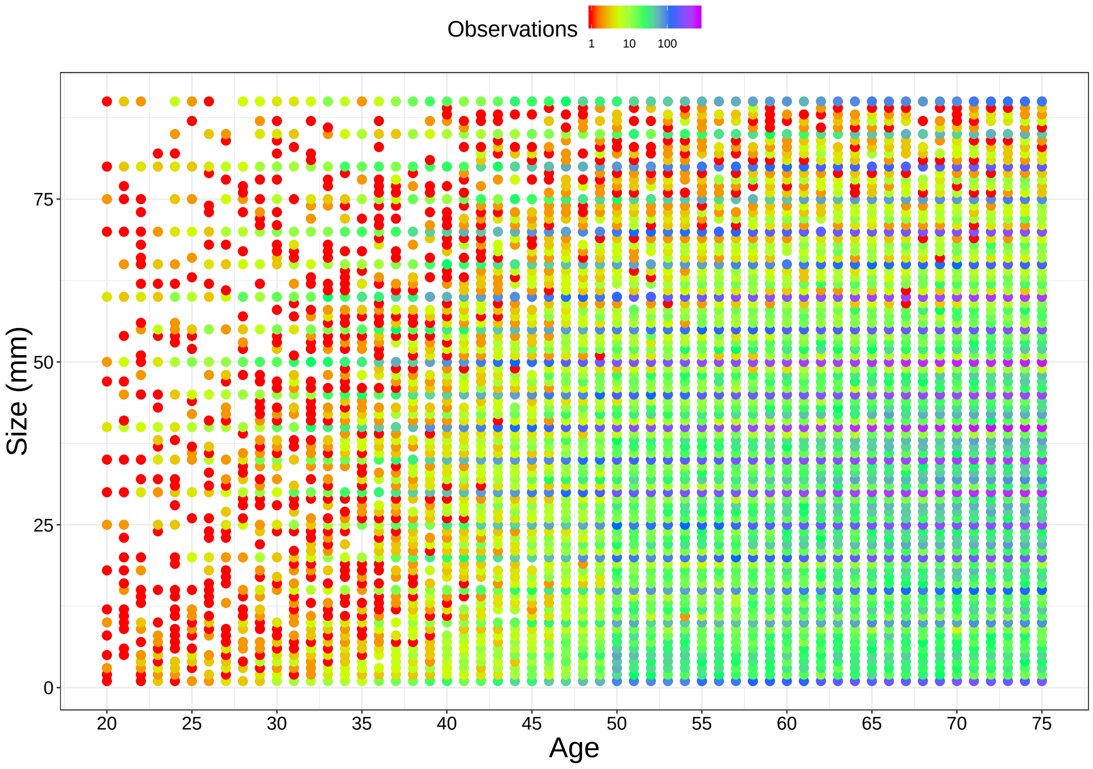

References

[1] Antal T, Krapivsky PL. Exact solution of a two-type branching process: models of tumor progression. Journal of Statistical Mechanics: Theory and Experiment. 2011;2011(08):P08018. doi:10.1088/1742-5468/2011/08/P08018.
